# Characteristic of the gene candidate *SecARS* encoding alkylresorcinol synthase in *Secale*

**DOI:** 10.1007/s11033-023-08684-y

**Published:** 2023-08-24

**Authors:** Małgorzata Targońska-Karasek, Michał Kwiatek, Jolanta Groszyk, Jakub Walczewski, Mariusz Kowalczyk, Sylwia Pawelec, Maja Boczkowska, Anna Rucińska

**Affiliations:** 1 Polish Academy of Sciences Botanical Garden—Center for Biological Diversity Conservation in Powsin, Warszawa, Poland; 2 Department of Genetics and Plant Breeding, Poznań University of Life Sciences, Poznań, Poland; 3 Plant Breeding and Acclimatization Institute (IHAR), National Research Institute, Radzików, Poland; 4 Institute of Soil Science and Plant Cultivation, State Research Institute, 24-100 Puławy, Poland

**Keywords:** Alkylresorcinols, Alkylresorcinol synthase, *Secale*, Rye, Gene

## Abstract

**Background:**

Alkylresorcinols (ARs) are compounds belonging to the class of phenolic lipids. A rich source of ARs are cereal grains such as rye, wheat, triticale or barley. ARs found in plants are characterized by a variety of biological properties such as antimicrobial, antifungal and cytotoxic activity. Moreover, they are proven to have a positive influence on human health. Here, we aimed to find and characterize the gene with ARs synthase activity in the species *Secale cereale*.

**Methods and results:**

Using BAC library screening, two BAC clones containing the gene candidate were isolated and sequenced. Bioinformatic analyses of the resulting contigs were used to examine the structure and other features of the gene, including promoter, intron, 3’UTR and 5’UTR. Mapping using the FISH procedure located the gene on the 4R chromosome. Comparative analysis showed that the gene is highly similar to sequences coding for type III polyketide synthase. The level of gene expression in various parts of the plant was investigated, and the biochemical function of the gene was confirmed by heterologous expression in yeast.

**Conclusions:**

The conducted analyses contributed to a better understanding of the processes related to ARs synthesis. Although the research concerned the rye model, the knowledge gained may help in understanding the genetic basis of ARs biosynthesis in other species of the Poaceae family as well.

**Supplementary Information:**

The online version contains supplementary material available at 10.1007/s11033-023-08684-y.

## Introduction

5-n-alkylresorcinols (1,3-Dihydroxy-5-alkylbenzenes, alkylresorcinols, ARs), also called resorcinolic lipids, are members of a broad family of chemical compounds that possess varied bioactivities and biological roles, related to phenolic lipids [1]. ARs are formed during secondary metabolic reactions. Hydrophilic resorcinol rings derived from polyketide-associated biosynthesis pathways, and hydrophobic alkyl chains are biosynthesized during fatty acid production. ARs have a characteristic odd carbon chain whose length depends on the lipid origin [2].

To date, about 150 naturally existing ARs have been characterized. The presence of these resorcinolic lipids has been demonstrated in 12 families of higher plants, such as: Poaceae, Anacardiaceae, Proteaceae, Myristicaceae, Ginkgoaceae, and Leguminoseae [3]. They have been identified in some mosses, lichen, fungi, and algae [4]. ARs have also been also found in prokaryotic organisms [5]. In the case of plants, accumulation of ARs occurs mainly within grains of cereals such as wheat, rye, triticale and barley, existing within a thin cuticle layer external to the seed coat. ARs have also been found to be concentrated within the cuticle of rye leaves, with similar homolog compositions occurring on the adaxial and abaxial leaf surfaces [6]. The root systems of *Oryza* spp. exude ARs mixtures, and likewise, *Sorghum* spp. exudates contain the ARs derivative sorgoleone [1, 6, 7].

ARs are distinguished by many biological properties, such as antibacterial activity, cytotoxic activity, antimutagenic activity, DNA and RNA synthesis inhibiting properties, inhibiting of enzymes, and interaction with biological membranes by incorporation to the membrane structure [8]. The high content of ARs in cereal grains most likely protects them from pathogens during the germination process, ensuring the effective survival of seeds during the dormant period. It has also been discovered that ARs, when consumed with products of plant origin, have a beneficial effect on human health [9]. By inhibiting the activity of the ω-tocopherol hydrolase enzyme, ARs contribute to an increase in the amount of the biologically active form of vitamin E (γ-tocopherol). This, in turn, reduces the LDL cholesterol fraction in the blood, and thus reduces the risk of developing cardiovascular diseases [8, 10]. Due to their functions, as well as resistance to heat treatment, ARs have been proposed as biomarkers of whole grain intake [9–11].

At present, the information about the enzymes involved in the synthesis of ARs in rye is rather poor. The knowledge on enzymes involved in the ARs synthesis in other plants is also incomplete, particularly with regard to genetic background. To date, data concerning genes encoding proteins with alkylresorcinol synthase (ARS) activity in Poaceae is limited to only three species: *Oryza sativa*, *Sorghum bicolor* and *Secale cereale*. In the case of *Oryza sativa* and *Sorghum bicolor*, only the coding sequences of ARS genes have been described [1, 6]. The enzyme encoding for AR synthase in *Secale cereale* described in the work of Sun et al. [12] is 43.4 kD and is encoded by a 1.23 kb cDNA. The sequence of the gene consists of two exons and one intron. Thus, there is no information about full sequences of ARS genes containing regulatory regions, such as promoter and UTR regions, in the existing literature. The aim of this study was to obtain full sequences of ARS genes, including exons, introns, promoters and UTR regions. The proposed research will also result in the acquisition of knowledge about the chromosomal localization of ARS genes. To date, such analyses have not been conducted in rye.

## Materials and methods

### DNA isolation

Plant material consisted of four rye forms: the inbred line L318 from the Department of Plant Genetics, Breeding and Biotechnology at the Warsaw University of Life Sciences in Warsaw, *Secale strictum* subsp. *africanum* from the Polish Academy of Sciences Botanical Garden - Center for Biological Diversity Conservation in Powsin, the Dańkowskie Złote cultivar from the Danko breeding company (Poland), and Daniello F1 from KWS Lochow-Petkus (Germany). For DNA isolation, each rye form used in analyses was represented by 5 individual plants. Plants were grown in multitrays in an air-conditioned greenhouse. Two-week-old leaves were collected and lyophilized. Total DNA was isolated using a modified CTAB method [13]. Plasmid DNA from pooled BAC plates was isolated with the alkaline lysis method [14]. DNA quality and concentration were evaluated using a NanoDrop One (Thermo Scientific, Waltham, USA) spectrophotometer.

### Amplifying the rye genes encoding ARS

Based on the DNA fragments including sequences coding for ARS enzymes in sorghum and rice available in databases, primer pairs were designed using the Primer3 program and used for amplification in rye DNA. Amplicons were compared in ClustalW (United Kingdom). The resulting amplicons were sequenced and compared with the source sequences followed by designing rye specific primers. The rye-specific primers were used for screening a rye BAC library in order to pick out BAC clones containing the *SecARS* sequence. The sequences of the specific rye primers were as follows: forward primer – ATCTTCGCCGAGAACCTGTT; reverse primer – CCTCGTGGTCGTACAGGTC. PCRs were conducted in 15 µL volumes containing 50 ng of genomic DNA, 0.5 U of DreamTaq polymerase (Thermo Scientific, Waltham, USA), 1.5 x DreamTaq buffer, 0.2 mM dNTPs and 0.2 µM of each primer. Amplification was carried out in an Arktik Thermal Cycler (Thermo Scientific, Waltham, USA) under conditions specified by the polymerase producer. The amplified products were separated on 1% agarose gel.

### Isolation of *ScARS* genes from the BAC library

Construction of the rye BAC library was described previously [15]. The rye *SecARS* gene was isolated from the BAC library using rye-specific primers and the Amplicon Express strategy (http://ampliconexpress.com/products-services/screening-services/pools-and-superpools) as follows: 39 superpools, each containing 2688 individual BAC clones from 7 plates were prepared for the first round of PCR. The second PCR round was performed on the matrixed Plate, Row and Column pools from the selected Superpools. Finally, BAC clones containing the desired sequences were picked and sequenced (external service at Genomed S. A. in Warsaw, Poland).

### Bioinformatic analysis of the *SecARS* sequence

Bioinformatic analyses were performed to determine the full gene sequence and structure. Several computer programs were used: BioEdit for *SecARS* gene identification, SoftBerry/FGENESH (http://www.softberry.com) for assessing gene structure, and PlantCare (http://bioinformatics.psb.ugent.be/webtools/plantcare/html/) [16] for promoter analysis.

### Phylogenetic analyses

The obtained *SecARS* sequence was used as a query for a BLAST search in the NCBI database (http://www.ncbi.nlm.nih.gov/) to find orthologue sequences presenting in other species. DNA sequence alignment was conducted based on the neighbor-joining method using ClustalX2. Phylogenetic analysis and dendrogram construction was performed in Mega-X.

### Structure modeling of the SecARS protein

Based on the full coding sequence of the *SecARS* gene, the structure of the SecARS protein was modeled with I-TASSER, which utilizes a multiple-threading approach [17] .

### Mapping of *SecARS* on rye chromosomes

Accumulation and fixation of mitotic chromosomes were carried out according to Hasterok et al. [18]. Root tips collected from seedlings of the L318 inbred line of rye were incubated at 4 °C for 26 h in order to accumulate the metaphase chromosomes, followed by fixation in an ethanol:acetic acid solution (3:1 v/v). Digestion was performed in 0.2% (v/v) Onozuka R-10 and Calbiochem cytohelicase (1:1 ratio) and 20% pectinase (Sigma) in 10 mM citrate buffer (pH 4.6) at 37 °C for 2 h and 40 min. Metaphase chromosomes were prepared as described by Heckmann et al. [19] with minor modifications of heating temperature as reported by Kwiatek et al. [20].

A DNA fragment including 768 bp of the promoter region, a 5’UTR sequence, and the first exon and intron of the newly described gene (full length 1295 bp) was used in the labeling procedure. The primer pairs sequences used for amplifying this DNA fragment were as follow: forward: GAATACGGGGTTTACGCTGA and reverse: CTCGACATGCTGGTGCTCC. The obtained DNA fragment was labelled using Dig High Prime Kit (Roche). A pTa-86 clone characterized by Komuro et al. [21] was amplified from the genomic DNA of wheat (Chinese Spring) according to Kwiatek et al. [22], and labeled with the Nick translation kit (Roche) using tetramethyl-5dUTP-rodamine (Roche). This clone carries a pSc119.2 repetitive sequence [23], which is specific to the rye genome and enables all rye chromosomes to be distinguished.

The fluorescence in situ hybridization (FISH) procedure was described by Kwiatek et al. [22] with minor modifications. The slides were analyzed with the use of an Axio Observer 7 (Carl Zeiss, Oberkochen, Germany) fluorescence microscope. Image processing was done using ZEN Pro software (Carl Zeiss, Oberkochen, Germany).

For a double confirmation of our gene localization, we conducted blast analysis with rye genomic sequences using the Galaxy tool: https://galaxy-web.ipk-gatersleben.de/ [24]. The *SecARS* coding sequence was used as the query.

### RNA isolation and cDNA synthesis

RNA was obtained from dry seeds, 2-day germinated seeds and 2-week old leaves of the same four rye forms as in DNA isolation. Fresh tissue (100 mg) was ground in liquid N2, then total RNA was isolated using the GeneMATRIX Universal RNA Purification Kit version 1.2 (Eurx, Gdańsk, Poland) in accordance with the manufacturer’s protocol. The RNA integrity was verified by agarose electrophoresis and concentration was measured using a NanoDrop One spectrophotometer (Thermo Scientific, USA). One µg of isolated RNA was then used as a template for cDNA synthesis with the use of the Maxima H Minus First Strand cDNA Kit with dsDNAse (thermo Fisher Scientific, USA).

### Quantitative real time PCR analyses

Real-time PCR reactions were performed with the obtained cDNA using a model Rotor Gene 6000 (Corbett) with three biological and three technical replicates. Primers specific to the cDNA of *SecARS* genes were designed with the use of Primer3 software. Two genes were used as an internal control of the expression analyses: *HvAct* (GenBank, accession No. AY145451) and *Sc18sRNA* (GenBank, accession No. JF489233.1). The following program was used: 95 °C for 2 min; 40 cycles of 95 °C for 5 s, 65 °C for 10 s, and 72 °C for 15 s. The total volume of the reaction mixture was 10 µL, which contained 2 µL cDNA, 0.4 µL each gene-specific primer (10 µM), 2.2 µL RNase-free water, and 5 µL SensiFAST SYBR No-ROX Kit (Meridian Bioscience, USA). The qPCR reaction efficiency for primers used in the analysis ranged from 0.365 to 0.692 for analyses with *Sc18sRNA* as reference, and 0.571 to 1.165 with *HvAct* as reference. The R2 value was ≥ 0.99. The stability of the reference genes was estimated based on C_T_ values. The obtained data were analyzed using the standard curve method [25].

### Heterologous expression in yeast

Full-length sequences of *ScARS* were generated using primers with an *Eco*RI restriction site, the KAPA HiFi PCR Kit, and the cDNA of the L318 rye inbred line as a template. PCR was carried out in a mixture containing 5 µL 5× KAPA HiFi GC Buffer, 0.75 µL KAPA dNTP (10 mM), 0.75 µL Forward Primer (10 µM), 0.75 µL Reverse Primer (10 µM), 0.5 µL KAPA HiFi (1 U/µL), 2 µL cDNA using the Verity 96 Thermal Cycler and the following profile: an initial denaturation, 3 min, 95 °C; 35 cycles of amplification: denaturation, 20 s, 98 °C; annealing, 15 s, 60 °C; and extension, 60 s, 72 °C; the final extension step, 1 min, 72 °C; and cooled, 4 °C.

The 25 µL of PCR and 5 µg of pGAP2 were used for restriction digestion with *Eco*RI enzyme in a total volume of 50 µL. The reaction was carried out in a mixture containing 1 µL *Eco*RI enzyme, 5 µL 10 x *Eco*RI buffer, and water to a total volume of 50 µL. The *ScARS* insert was purified using the PCR/DNA Clean-Up Purification Kit (EURx, Gdańsk, Poland). Linearized plasmid was dephosphotrylated with Fast AP (2 µL 10× Fast AP Buffer, 2 µL Fast AP (1 U/µl), and 16 µL nuclease-free water to a total volume of 40 µL). The reaction was incubated for 40 min at 37 °C, then for 5 min at 75 °C for enzyme inactivation. Linearized plasmid was purified using the PCR/DNA Clean-Up Purification Kit according to the manufacturer’s protocol.

Ligation of the cDNA insert into linearized pGAP2 was performed using T4 DNA Ligase (New England BioLabs, Frankfurt, Germany) with 2 µL 10× T4 DNA Ligase Buffer, 10 µL linearized pET-21d (+) vector (50 ng), 4.2 µL cDNA insert (60 ng), 1 µL T4 DNA Ligase, and water to a final volume of 20 µL. Samples were incubated overnight at 16 °C, 10 min at 65 °C, and cooled to 4 °C. Five µL of the reactions were used for *E. coli* JM107 (Fermentas, Vilnius, Lithuaina) competent cell transformation by the heat shock method. Transformed JM107 were cultured and selected on LB medium with ampicillin (100 mg · L^-1^) (A&A Biotechnology, Gdynia, Poland). After chloroform extraction and isopropanol precipitation, the insert was digested with the use of *Avr*II (New England BioLabs, Frankfurt, Germany) and forwarded for transformation in yeast.

The *Pichia pastoris* X33 wild strain was cultivated overnight in 25 ml of liquid YPD medium in a 100 ml flask in 30 °C on an orbital shaker set for 250 rpm. Twenty µL were used to inoculate 100ml of fresh YPD medium in a 1 l bottle, and cultivated at 30 °C on a 250 rpm orbital shaker until OD_600_ reached 1–2.

The number of cells were calculated according to the formula: 1A_600_ = 5 × 10^7^. For one transformation, 8 × 10^8^ cells were centrifuged in a 50 ml sterile falcon tube at 500 G for 5 min.

Supernatants were discharged and cells were suspended in 10 ml of 100 mM LiAc (lithium acetate), 10 mM DTT, 0.6 M sorbitol, and 10 mM Tris-HCL pH 7,5.

Suspensions were incubated for 30 min at RT. Cells were pelleted, 1 ml of ice cold 1 M sorbitol was added. Suspensions were transferred to a 2 ml eppendorf tube. The cells were washed three times with ice cold 1 m sorbitol. After the final wash, 40 µl of ice cold sorbitol was added to the pellets.

One µg of linearized plasmids were mixed with the cells and the solutions were transferred to electroporation corvettes (2 mm gap) and incubated for 5 min.

An electroporating pulse was applied at 1.5 kV, 25 µF, 186 Ω. Next, the cells were diluted with 1ml ice cold 1 M sorbitol and transferred to 15 ml falcon tubes. The cells were incubated for 4 h at 30 °C.

After incubation, the cells were gently suspended and 100 µl, 400 and 500 µl were spread on a 9 cm petri dish with YPDS medium with 500 µg/ml Zeocin.

Plates were incubated at 30 °C upside down. Colonies were counted after 72 h, and replanted on YPD with 100 ug/ml of Zeocin.

### Biochemical analyses

For quantitative analyses, 500 mg samples of powdered plant material and lyophilized transformed yeasts were used. In the case of plant material, dry seeds, 2-day germinated seeds and 2-week-old leaves of the same four rye forms used before were analyzed. Dry seeds and leaves of the L318 inbred line were excluded from the analyses due to insufficient amounts of necessary material. The yeast material consisted of 6 transformed *Pichia pastoris* colonies and 3 untransformed controls. Extractions of plants and yeast samples were conducted with acetone in an ultrasonic bath at 20 °C for 48 and 24 h respectively. Additionally, in yeast samples, 20 µg of the internal standard, 4-dodecylresorcinol was added during extraction. The extracts were centrifuged (room temperature, 5 min, approx. 23 000 x g ), and supernatants were evaporated to dryness under reduced pressure. Plant extracts were re-dissolved in 1 ml of acetonitrile and filtered using centrifugal filters (leaf samples, 0.22 μm, Merck) or low evaporation PTFE filtering vials (grain samples, 0.2 μm, Thomson). Yeast extracts were re-dissolved with 2-propanol and stored at -20 °C. Prior to the LC-MS analyses, yeast samples were concentrated with a stream of N2 at room temperature, re-dissolved with 50 µl of 2-propanol, and filtered using centrifugal filters (regen. cellulose membrane with 0.22 μm pores, Merck).

High-resolution LC-MS analyses were conducted with a Thermo Scientific Ultimate 3000 RS chromatographic system hyphenated to a Bruker Impact II HD (Bruker, Billerica, USA) quadrupole-time of flight (Q-TOF) mass spectrometer. Chromatographic separations were carried out on a Waters CORTECS C8 column (2.1 × 100 mm, 2.5 μm, Milford, USA). The mobile phase A consisted of distilled water containing 0.1% (v/v) formic acid and 1% (v/v) 1 M ammonium acetate. The mobile phase B consisted of a mixture of 2-propanol and acetonitrile (7:3) containing 0.1% (v/v) of formic acid and 1% 1 M ammonium acetate.

A flow splitter was used to divert the column effluent at approx. 0.2 ml/min into the APCI (atmospheric pressure chemical ionization) ion source of the mass spectrometer. The critical parameters of the ion source were the following: capillary voltage 4 kV; corona current set to 6 µA; nebulizer gas (N2) pressure at 2.5 bar; drying gas (N2) flow at 3.0 l/min; drying temperature at 250 °C, and APCI heater at 220 °C. Argon was used as the collision gas. The MS/MS collision energy was automatically set between 2.5 and 35 eV. Ion transfer parameters were optimized for the m/z range from 100 to 800. In the case of plant samples, automatic internal mass calibration of the data was performed with a solution of APCI-L QTOF tune mixture (Agilent), which was diluted (1:4 v/v) with 50% 2-propanol and introduced into the ion source via a 20 µl loop at the beginning of each analysis. For yeast samples, automatic internal mass calibration of the data was performed with a 5% solution (w/v) of polyethylene glycol 4000 (PEG 4000) in 50% (v/v) 2-propanol and introduced into the ion source via a 20 µl loop at the beginning of each analysis.

After data acquisition and calibration, a set of ion chromatograms for the protonated molecules of known ARs was extracted from the full scan data with 0.005 Da width. Data acquisition and processing were carried out using Bruker Data Analysis software version 4.4 SR1.

Calibration curves were prepared using a series of dilution from stock solutions (1 mg/ml) of 5-pentadecylresorcinol and 5-eicosylresorcinol for plant samples and only 5-pentadecylresorcinol for yeasts samples. Concentrations of the ARs in the samples were calculated using the 5-eicosylresorcinol calibration curve. The MS response for 5-pentadecylresorcinol was linear in the concentration range from 15 to 200 ng/µl, with a limit of detection calculated at 4.6 ng/µl, respectively.

## Results

### Isolation of positive BAC clones

Using PCR with rye *SecARS* gene-specific primers, two clones were isolated from the rye inbred line L318 BAC library. The clones were sequenced and the resulting reads were aligned in contigs. Altogether, 233 contigs were arranged: 138 in the first and 95 in the second BAC clone. The desired *SecARS* sequence was identified in both selected clones. The isolation efficiency was 0.0007% and the presence of a given *SecARS* gene in more than one BAC clone indicated that *SecARS* is present in the rye genome as a single-copy gene.

### *SecARS* gene structure

**Fig. 1:**
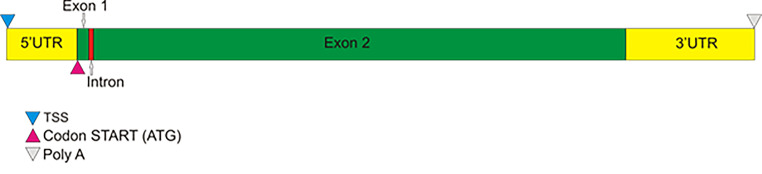
Graphical structure of *SecARS* gene

The predicted structure and length of the *SecARS* gene is presented in Tables 1 and Fig. 1. The gene is composed of 2 exons and 1 intron. The length of exons were 217 and 1013 base pairs (bp), respectively. The length of the intron was 91 bp. The predicted 5’ UTR started from the TSS region, 134 bp before the codon START (ATG), whereas 3’UTR was 245 bp long. The full sequence of predicted *SecARS* gene was deposited in the NCBI database with the number MH513639.

**Table 1:** Structural features of *SecARS* gene

Gene name	Acc. No. (NCBI)	Gene lenght (bp)	Exons No/total lenght	Intrron No/total lenght	Lenght of 3’UTR (bp)	Lenght of 5’UTR (bp)
*SecARS*	MH513639	1321	2/1230	1/91	245	134

### Phylogenetic analysis of *SecARS*

**Fig. 2:**
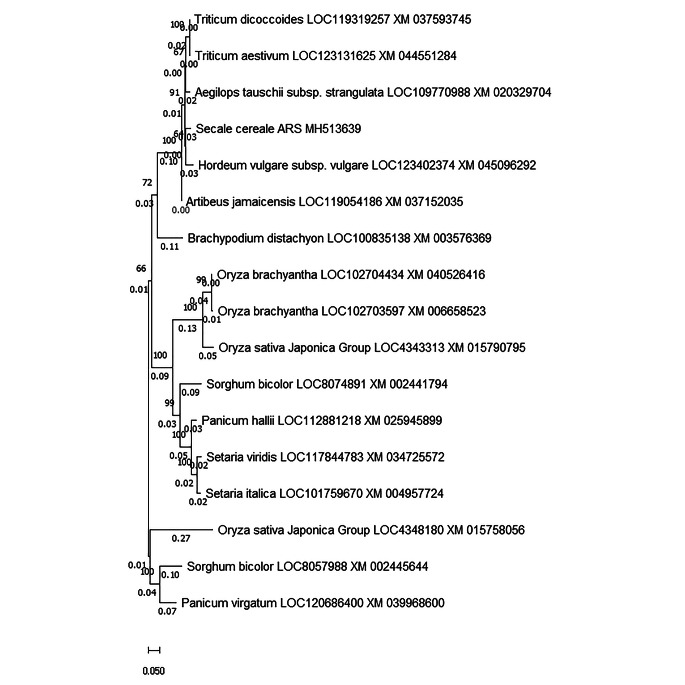
The phylogenetic tree constructed using the Neighbor-Joining method showing relations between 17 cds sequences. The bootstrap consensus tree inferred from 500 replicates. The percentage of replicate trees in which the associated taxa clustered together in the bootstrap test (500 replicates) are shown next to the branches. The evolutionary distances were computed using the Maximum Composite Likelihood method

Seventeen cDNA sequences were taken after a BLAST search in the NCBI database for sequence alignment (Fig. 2). The criteria for selection of accessions for dendrogram construction was the same family or high sequence similarity. A dendrogram based on the Neighbor-Joining method showed that the *SecARS* sequence has the highest similarity to *Hordeum vulgare* subsp. *vulgare* bisdemethoxycurcumin synthase-like sequence (XM_045096292). The genetic distance calculated using the Maximum Composite Likelihood model in this case was 0.059.

### Result of protein structure prediction

**Fig. 3:**
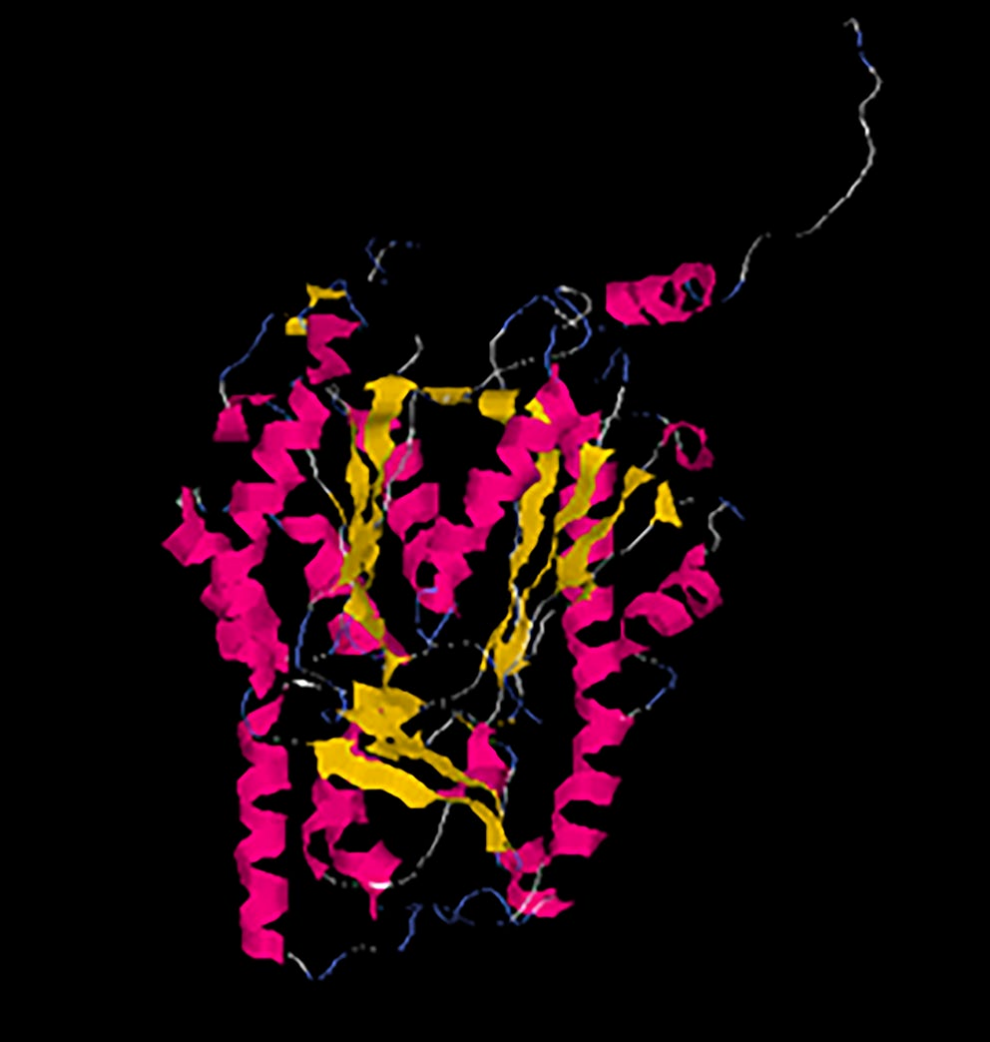
Structure of *SecARS* gene predicted by I-TASSER. The image shows the most likely protein structure based on the amino acid sequence. The function predicted by I-TASSER for this protein is type III polyketide synthase

Protein prediction by I-TASSER showed that the SecARS protein is 409 aminoacids long. The predicted secondary structure indicated that the protein contains helixes, strands and coils in its structure. The most probable final model predicted by I-TASSER is shown in Fig. 3. and the secondary structure is shown in **Online Resource 1**. The confidence of the proteins models model is quantitatively measured by a C-score that is calculated based on the significance of threading template alignments and the convergence parameters of the structure assembly simulations. The C-score of the model with the highest confidence was 0.51, signifying relatively high confidence, as the C-score is typically in the range of -5 to 2. The prediction indicated that this is a cytoplasmic protein involved in the chemical reactions and pathways resulting in the formation of flavonoids. Type III polyketide synthases were indicated as the closest structural similarity to SecARS.

### Promoter analysis

For analysis of the promoter region, the DNA fragment of 1522 bp before the START codon was arbitrarily selected. In this region, 115 different motifs were found (**Online Resource 2**). The most frequent were TATA-box (41% of all motifs) and CAAT-box elements (23% of all motifs). Stress-specific fragments potentially involved in abiotic stress response like light and low temperature were also found in the analyzed DNA fragment of the gene promoter. The promoter binding sites MYB and MYBHv1were also identified.

### Mapping of *SecARS* on rye chromosomes

**Fig. 4:**
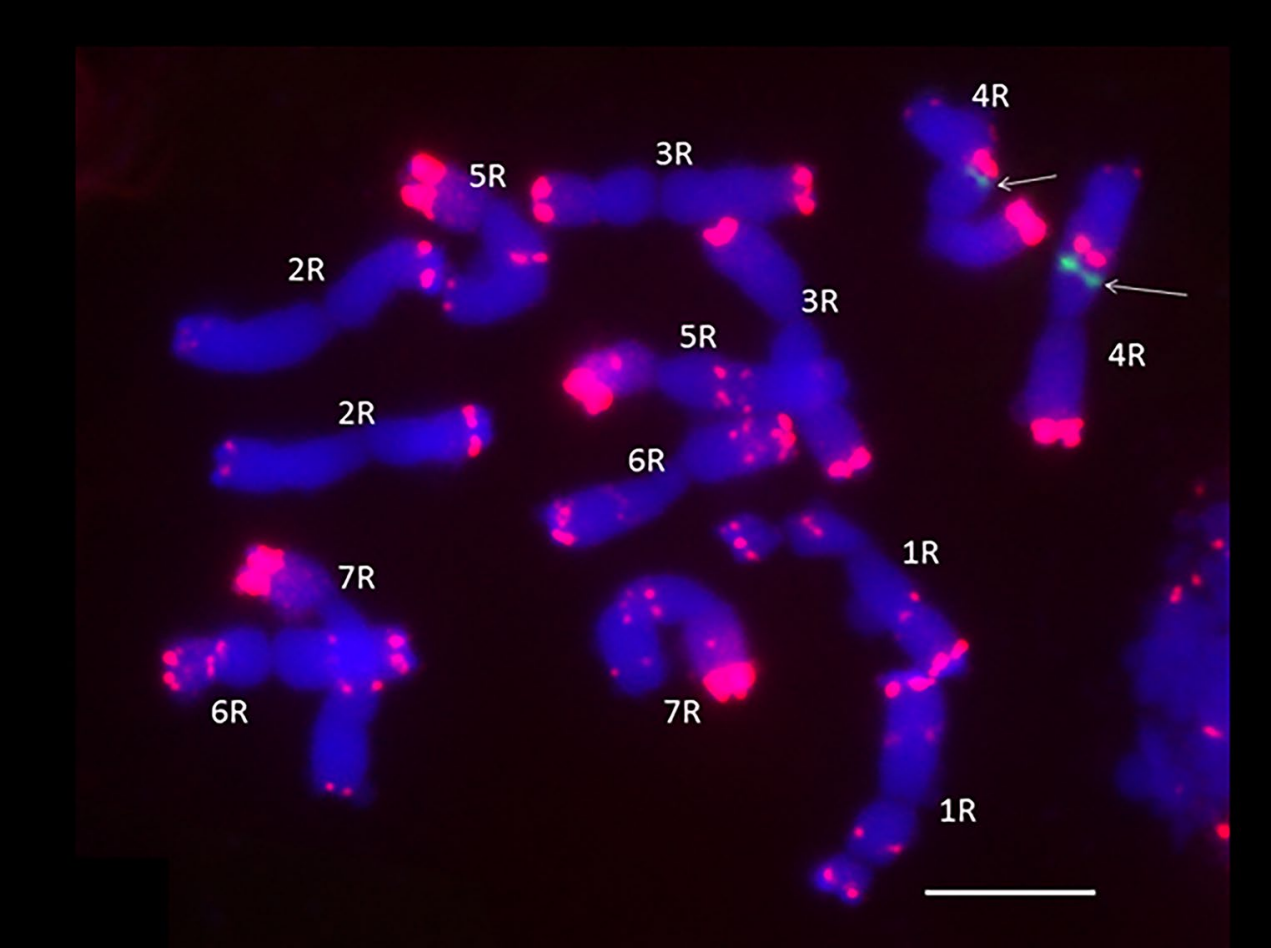
Rye L318 inbred line metaphase chromosomes. The arrows indicate the localization of the gene

After applying the procedure, signals were obtained on the long arm of the 4R chromosome. Signals were visible both in metaphase chromosomes and interphase nuclei (Fig. 4.). BLAST analysis with the *Secale cereale* genome confirmed the gene localization on the 4R rye chromosome.

### Expression of *SecARS* in different rye samples

**Fig. 5:**
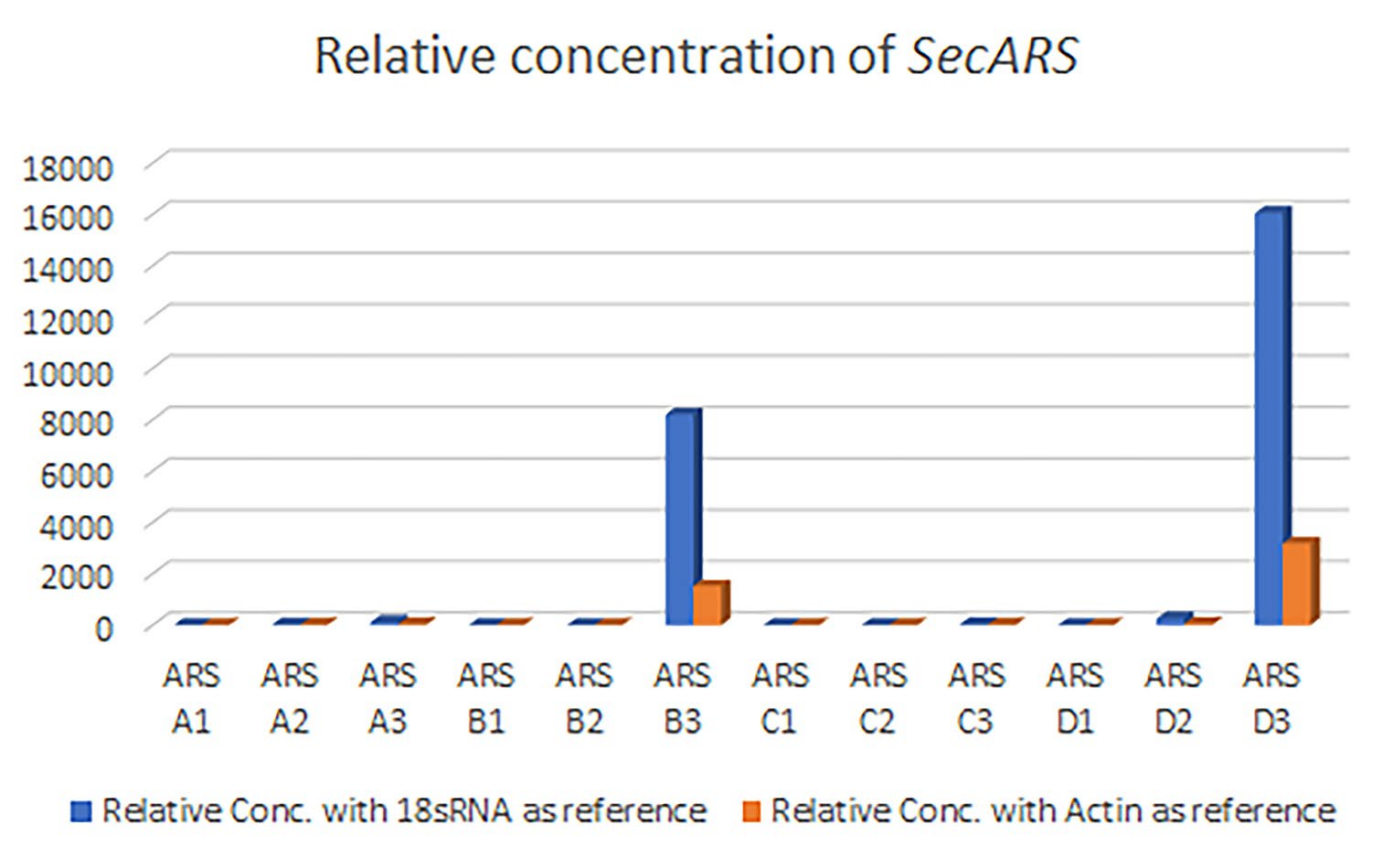
Relative concentration of expressed *SecARS* gene in different plant material. The symbols correspond accordingly: A1-Dańkowskie Złote dry seeds, A2-Dańkowskie Złote 2-day germinated seeds, A3-Dańkowskie Złote leaf, B1-L318 dry seeds, B2-L318 2-day germinated seeds, B3-L318 leaf, C1-*S. strictum* dry seeds, C2-*S. strictum* 2-day germinated seeds, C3-*S. strictum* leaf, D1-F1 Daniello dry seeds, D2-F1 Daniello 2-day germinated seeds, D3-F1 Daniello leaf

The expression profile of the *SecARS* gene varied in different rye forms and plant materials. Relative gene concentration was the highest in 2-week-old leaves. However, the level of expression was varied according to the cultivation status of the examined material. The highest relative concentration was observed in 2-week-old leaves of the Daniello hybrid cultivar and the inbred line L318. The relative concentration of *Secale strictum* and the Dańkowskie Złote leaf samples was much lower than in the other two rye forms. No expression was observed in dry seeds. For two-day-old germinated seeds, the expression was detectable, but very low in a comparison with leaves (Fig. 5, **Online resource 3**).

### Biochemical analyses

**Fig. 6:**
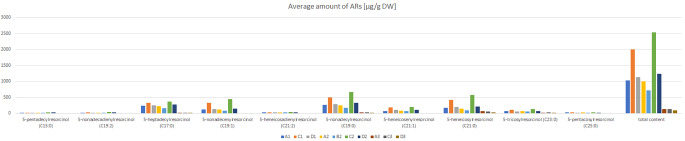
Content of ARs in different plant material. The symbols correspond accordingly: A1-Dańkowskie Złote dry seeds, A2-Dańkowskie Złote 2-day germinated seeds, A3-Dańkowskie Złote leaf, B1-L318 dry seeds, B2-L318 2-day germinated seeds, B3-L318 leaf, C1-*S. strictum* dry seeds, C2-*S. strictum* 2-day germinated seeds, C3-*S. strictum* leaf, D1-F1 Daniello dry seeds, D2-F1 Daniello 2-day germinated seeds, D3-F1 Daniello leaf

**Fig. 7:**
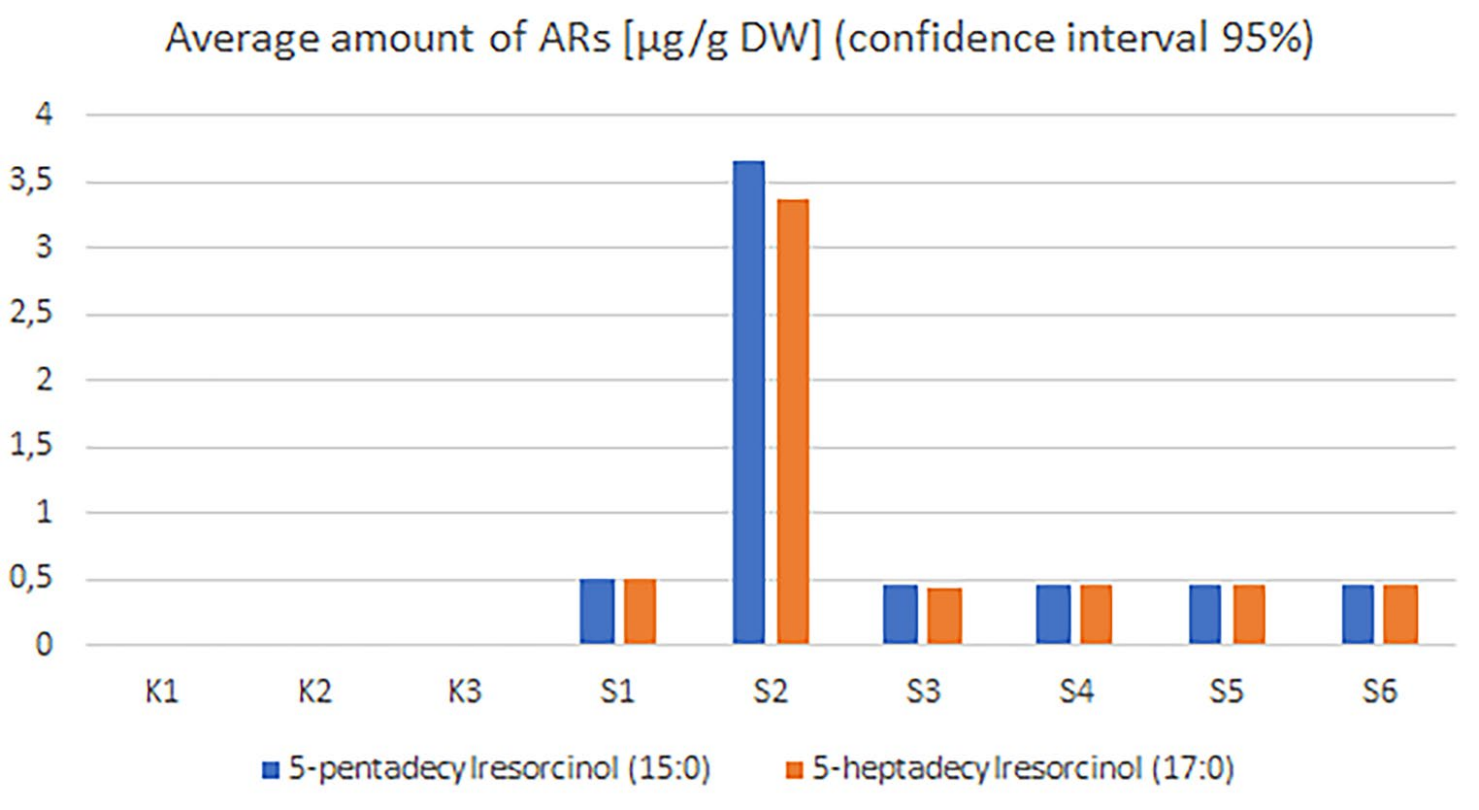
Average amount of ARs in *Pichia pastoris* samples. K1 to 3 are negative controls, S1 to 6 are transformed samples

In the case of plant materials, the highest amounts of ARs were detected in 2-day germinated seeds and dry seeds of *Secale strictum*, which was 2541.6 and 1997.9 µg/g dry weight (DW). Relatively high amounts of ARs were detected in 2-day germinated seeds and dry seeds of the hybrid cultivar Daniello: 1236.6 and 1133 µg/g DW respectively. The lowest amounts of ARs were detected in 2 week-old leaves of hybrid cultivar Daniello (89.4 µg/g DW). The most common homologs which occurred in the highest amounts were 5-nonadecylresorcinol (C19:0) and 5-heneicosylresorcinol (C21:0) (Fig. 6, **Online resource 4**). In order to confirm the function of the analyzed gene, we used gene transformation and heterologous expression in *Pichia pastoris*. In all of yeast samples including transformed *P. pastoris*, amounts between 0.46 and 3.65 µg/g DW of 5-pentadecylrezorcynol (15:0) and between 0.45 and 3.38 µg/g DW of 5-heptadecylrezorcynol (17:0) were detected. No ARs were detected in the untransformed samples (Fig. 7, **Online resource 5**).

## Discussion

At present, the information about enzymes involved in the synthesis of ARs in rye is rather poor. The knowledge on enzymes involved in ARs synthesis in other plants is also incomplete, particularly with regard to genetic background. In the presented study, we obtained a full sequence of *SecARS*, including exons, introns, promoters and UTR regions. We also found the chromosomal localization of the gene. To date, such analyses have not been conducted in rye.

When it comes to rye (*Secale cereale*), there is only one communication dealing with the genetic background of ARS where the putative gene coding for ARS was described [12]. The authors used a homology-based cloning approach and the Rapid Amplification of cDNA Ends (RACE) technique to amplify the putative gene encoding protein with ARS activity (herein after referred to as ARS genes) in *Secale cereale*. As a final result of these examinations, a cDNA represented by an open reading frame of 1.23 kb (corresponding to the sequence comprised of two exons separated by one intron) was found. The protein encoded by this sequence was 43.4 kD. The experiment on expressional activity employing the yeast model confirmed its ARS activity. However, neither the nucleotide sequence of the gene nor the 3’UTR, 5’UTR or promoter regulatory sequences have been published. Our results confirmed the sequence and structure of the *SecARS* gene composed of 2 exons and one intron. Moreover, we found a 134 bp long 5’UTR region which started from the TSS region, as well as a 245 bp long 3’UTR with a poliA region at the end. Our promoter analysis showed that in this relatively short region, there are 115 motifs involved in the transcription initiation process. Nevertheless, the analyzed region was established arbitrarily, so the real number of regulatory motifs in promoter region may be different. This is especially true because during analysis of contigs with the *SecARS* sequence, we found that there were no other coding sequences over 6 kbp upstream of our gene.

Mapping on rye chromosomes showed the localization of the *SecARS* gene on the long arm of the 4R chromosome. Chromosome 4R is known to contain QTLs for alpha-amylase activity, preharvest sprouting, kernel thickness, heading time, chlorophyll content in leaves, and flag leaf length [26]. In the work of Bolibok-Brągoszewska et al. [27], it was suggested that this chromosome contains regions that were subjected to selection pressure during domestication and might reflect the presence of genomic regions with limited polymorphism, possibly resulting from selection for QTLs located therein and controlling adaptive traits and quality characters relevant for cultivation in Central and Northern Europe. This thesis was supported by a relatively high genetic similarity average value for chromosome 4R deviating from the general pattern of differences in chromosome specific average GS values observed within germplasm groups [27]. Not without significance could be the fact that for years rye breeding was focused on producing cultivars with the lowest ARs content in grains [28].

The high similarity of our predicted protein to type III polyketide synthase (PKS) is in agreement with other works describing ARS in *Secale* [12]. Plant ARSs are members of the type III PKS family of proteins, a large group of enzymes that produce a wide array of secondary metabolites. A type III PKS that produces 5-n-alkylresorcinols from fatty acyl-CoA starter units, called ARS has also been described in several microorganisms including *Azotobacter vinelandii*, *Streptomyces griseus* and *Neurospora crassa*, as well as mosses like *Physcomitrella patens* [29]. These enzymes occur as homodimers possessing subunits between 40 and 45 kD in size and catalyze iterative decarboxylative condensation reactions [2, 5, 7]. Apart from ARS, additionally stilbene synthase (STS) and chalcone synthase (CHS) are enzymes that belong to this family [6]. All PKSs utilize a starter unit and perform three condensation reactions with malonyl-CoA as the extender unit, yielding a tetraketide intermediate. The details of these reactions attracted much interest about 20 years ago, as PKSs involved in the biosynthesis of aromatic ring-containing intermediates mainly utilize either an aldol or a Claisen condensation-based mechanism for ring folding [30]. ARSs use an STS-type cyclization mechanism [1, 5]. This distinction among ring folding mechanisms provides very useful information about the evolutionary history of ARSs and PKSs in general [6].

Our results confirmed that the composition and amount of Ars differs depending on the species and part of the plant. In the work of Ross et al. [31], it was proved that the composition of homologs is constant for each species of cereal but it can be different for each particular variety. In rye seeds, this content fluctuated between 360 and 3200 µg/g DW and this was much more than in wheat seeds, where this content is 317–1430 µg/g DW [8]. In the presented work, the highest content of ARs in cereal seeds was in the wild species *Secale strictum* (1997.9 µg/g DW), whereas the lowest content was detected in the rye cultivar Dańkowskie Złote (1035.6 µg/g DW). These differences between wild and cultivated rye may result from the fact that over the years of cultivation and breeding, rye forms with the lowest ARs content in grains were preferred and selected by breeders, because ARs were attributed to anti-nutritional properties [32]. In leaf samples, the ARs amount fluctuated between 136.9 µg/g DW in the case of Dańkowskie Złote to 89.4 µg/g DW for the Daniello cultivar. However, these differences may be due to genetic and environmental factors [31]. Genetic and environmental factors may also be related to the different ARs homolog content. In our work, the most common homologs, were C19:0 and C21:0, whereas in the work of Sun et al. [12], C23:0 and C21:1 also occurred. Moreover, our results are also in contrast to previous literature showing that ARs on different rye organs had side chain lengths ranging only from C19 to C23 [12, 33], which suggested a narrow range of substrates to the enzyme in rye. In our work, relatively smaller amounts of C15:0 AR and C17:0 were detected in plant samples. On the other hand, the results of heterologous expression in *Pichia pastoris* showed that transformed yeast can produce small amounts of ARs. Only C15:0 AR and C17:0 were detected. This result does not confirm the previous literature reports [12], where AR synthase transformed to *Saccharomyces cerevisiae* accepted diverse substrate chain lengths to produce a broad series of homologous ARs. However, in our study a different yeast species was used in the analyses.

Analysis of gene expression showed that the highest expression level was in the case of leaf tissue, which was in agreement with the work of Sun et al. [12]. Moreover, similarly to Sun et al. [12], no expression was observed in rye seed samples. Furthermore, we did not observe a correlation between the level of gene expression and the amount of ARs in plant tissues, which can suggest that rye ARs are mostly produced in rye leaves, and then transported to other organs.

Statements & Declarations.

### Electronic supplementary material

Below is the link to the electronic supplementary material.

Supplementary Material 1

Supplementary Material 2

Supplementary Material 3

Supplementary Material 4

Supplementary Material 5

## Data Availability

DNA sequence was deposited in the NCBI database by the number MH513639. Other data generated or analyzed during this study are included in this published article (and its supplementary information files). Research involving human participants and/or animals - This article does not contain any studies with human participants or animals performed by any of the authors.
