# Supplementary material for: Characteristic of the gene candidate SecARS encoding alkylresorcinol synthase in Secale
Source: Mol Biol Rep. 2023 Aug 24;50(10):8373–83. doi: 10.1007/s11033-023-08684-y (PMC10520190; doi:10.1007/s11033-023-08684-y)

Online Resource 1. Predicted by I-TASSER normalized B factor with the secondary structure of SecARS protein.


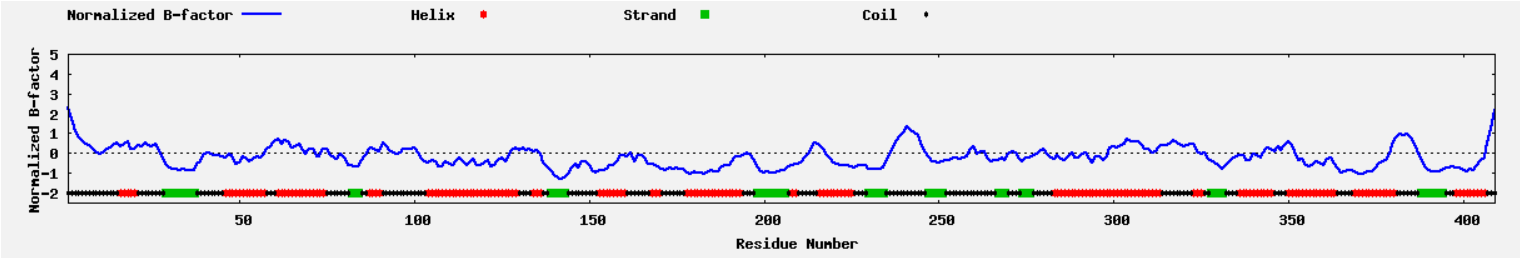

Supplement: Supplementary file 1 — Supplementary Material 1 [file 11033_2023_8684_MOESM1_ESM.docx]
