# Supplementary material for: Characteristic of the gene candidate SecARS encoding alkylresorcinol synthase in Secale
Source: Mol Biol Rep. 2023 Aug 24;50(10):8373–83. doi: 10.1007/s11033-023-08684-y (PMC10520190; doi:10.1007/s11033-023-08684-y)
Supplement: Supplementary file 3 — Supplementary Material 3 [file 11033_2023_8684_MOESM3_ESM.docx]

Online Resource 2. Relative concentration of expressed *SecARS* gene in different plant material

| Replicate Name/Plant material | Relative Conc. with 18sRNA as reference | Relative Conc. with actin as reference |
| --- | --- | --- |
| A1 / Dańkowskie Złote dry seeds | 0.000267 | 0.000067 |
| A2 / Dańkowskie Złote 2-day germinated seeds | 9.0571 | 1.60173 |
| A3 / Dańkowskie Złote leaf | 111.82063 | 21.69023 |
| B1 / L318 dry seeds | 0.0058 | 0.0009 |
| B2 / L318 2-day germinated seeds | 0.07253 | 0.01555 |
| B3 / L318 leaf | 8184.9652 | 1495.0539 |
| C1 / *S. strictum* dry seeds | 0.0003 | 0.00012 |
| C2 / S. strictum 2-day germinated seeds | 0.3125 | 0.05395 |
| C3 / *S. strictum* leaf | 20.582067 | 4.393 |
| D1 / F1 Daniello dry seeds | 0.0003 | 0.0001 |
| D2 / F1 Daniello 2-day germinated seeds | 246.1003 | 33.8823 |
| D3 / F1 Daniello leaf | 16034.4547 | 3154.3367 |
