# Supplementary material for: Characteristic of the gene candidate SecARS encoding alkylresorcinol synthase in Secale
Source: Mol Biol Rep. 2023 Aug 24;50(10):8373–83. doi: 10.1007/s11033-023-08684-y (PMC10520190; doi:10.1007/s11033-023-08684-y)
Supplement: Supplementary file 4 — Supplementary Material 4 [file 11033_2023_8684_MOESM4_ESM.docx]

Online Resource 3. Content of ARs in different plant material

| AR type | Average amount of ARs [µg/g DW] | | | | | | | | | |
| --- | --- | --- | --- | --- | --- | --- | --- | --- | --- | --- |
|  | A1 / Dańkowskie Złote dry seeds | C1 / *S. strictum* dry seeds | D1 / F1 Daniello dry seeds | A2 / Dańkowskie Złote 2-day germinated seeds | B3 / L318 leaf | C2 / S. strictum 2-day germinated seeds | D2 / F1 Daniello 2-day germinated seeds | A3 / Dańkowskie Złote leaf | C3 / *S. strictum* leaf | D3 / F1 Daniello leaf |
| 5-pentadecylresorcinol (C15:0) | 22,7 | 21,9 | 23,3 | 20,7 | 14,7 | 25,5 | 24,7 | 0 | 0 | 0 |
| 5-nonadecadienylresorcinol (C19:2) | 22,4 | 34,2 | 21,9 | 21,9 | 16,6 | 45,4 | 24,5 | 0 | 0 | 0 |
| 5-heptadecylresorcinol (C17:0) | 238,5 | 326,9 | 249,4 | 228,8 | 154,5 | 373,1 | 275,9 | 16,3 | 14,4 | 10,9 |
| 5-nonadecenylresorcinol (C19:1) | 118,5 | 328,4 | 131,4 | 115,6 | 87,3 | 440,3 | 152,3 | 0 | 0 | 0 |
| 5-heneicosadienylresorcinol (C21:2) | 32,6 | 36,3 | 26,1 | 29,1 | 24,5 | 39,5 | 26,8 | 0 | 0 | 0 |
| 5-nonadecylresorcinol (C19:0) | 258,3 | 494,2 | 284,2 | 254,6 | 175,8 | 668,2 | 323 | 33,4 | 26,5 | 19,9 |
| 5-heneicosenylresorcinol (C21:1) | 70,1 | 188,7 | 114,8 | 79,9 | 76 | 201,6 | 111,3 | 0 | 0 | 0 |
| 5-heneicosylresorcinol (C21:0) | 168,9 | 425,9 | 199,9 | 151,7 | 98,6 | 581,7 | 213,1 | 65,4 | 61,1 | 36,3 |
| 5-tricosylresorcinol (C23:0) | 67 | 114,9 | 60,8 | 63,4 | 51,1 | 137,5 | 65,6 | 21,8 | 31,5 | 22,3 |
| 5-pentacosylresorcinol (C25:0) | 36,6 | 26,5 | 21,2 | 35,2 | 20,6 | 28,8 | 19,4 | 0 | 0 | 0 |
| total content | 1035,6 | 1997,9 | 1133 | 1000,9 | 719,7 | 2541,6 | 1236,6 | 136,9 | 133,5 | 89,4 |
