# Supplementary material for: Characteristic of the gene candidate SecARS encoding alkylresorcinol synthase in Secale
Source: Mol Biol Rep. 2023 Aug 24;50(10):8373–83. doi: 10.1007/s11033-023-08684-y (PMC10520190; doi:10.1007/s11033-023-08684-y)
Supplement: Supplementary file 5 — Supplementary Material 5 [file 11033_2023_8684_MOESM5_ESM.docx]

Online Resource 4. Average amount of ARs in *Pichia pastoris* samples. K1 to 3 are negative controls, S1 to 6 are transformed samples.

| Sample | Average amount of ARs [µg/g DW] (confidence interval 95%) | |
| --- | --- | --- |
|  | 5-pentadecylresorcinol (15:0) | 5-heptadecylresorcinol (17:0) |
| K1 | 0 | 0 |
| K2 | 0 | 0 |
| K3 | 0 | 0 |
| S1 | 0.51 | 0.51 |
| S2 | 3.65 | 3.38 |
| S3 | 0.47 | 0.45 |
| S4 | 0.46 | 0.46 |
| S5 | 0.46 | 0.46 |
| S6 | 0.46 | 0.47 |
